# Progress and Prospects of Developing Climate Resilient Wheat in South Asia Using Modern Pre-Breeding Methods

**DOI:** 10.2174/1389202922666210705125006

**Published:** 2021-12-30

**Authors:** Sivakumar Sukumaran, Hari Krishna, Kuldeep Singh, Khondoker Abdul Mottaleb, Matthew Reynolds

**Affiliations:** ^1^ Global Wheat Program, International Maize and Wheat Improvement Center (CIMMYT), Mexico City, Mexico; ^2^Scioeconomics Program, CIMMYT, Mexico City, Mexico; ^3^Division of Plant Breeding and Genetics, Indian Agricultural Research Institute, New Delhi, India; ^4^National Institute of Plant Genetic Resources (NIPGR), New Delhi, India

**Keywords:** Climate change, pre-breeding, spring wheat, heat stress, drought stress, CIMMYT

## Abstract

Developing climate-resilient wheat is a priority for South Asia since the effect of climate change will be pronounced on the major crops that are staple to the region. South Asia must produce >400 million metric tons (MMT) of wheat by 2050 to meet the demand. However, the current average yield <3 t/ha is not sufficient to meet the requirement. In this review, we are addressing how pre-breeding methods in wheat can address the gap in grain yield as well as reduce the bottleneck of genetic diversity. Physiological pre-breeding which incorporates screening of diverse germplasm from gene banks for physiological and agronomic traits, the strategic crossing of complementary traits, high throughput phenotyping, molecular markers-based generation advancement, genomic prediction, and validation of high-value heat and drought tolerant lines to South Asia can help to alleviate the drastic effect of climate change on wheat production. There are several gene banks, if utilized well, can play a major role in breeding for climate-resilient wheat. CIMMYT’s wheat physiological pre-breeding has delivered several hundred lines *via* the Stress Adapted Trait Yield Nursery (SATYN) to the NARS in many South Asian countries; India, Pakistan, Nepal, Bangladesh, Afghanistan, and Iran. Some of these improved germplasms have resulted in varieties for farmer's field. We conclude the review by pointing out the importance of collaborative interdisciplinary translational research to alleviate the effects of climate change on wheat production in South Asia.

## 1 INTRODUCTION

In Asia, wheat is the second largest crop after rice and these two crops ensure food security of almost 57% of the world's population and livelihood of 80% of smallholder farmers of the world [1]. The production, productivity, and area *per se* have increased in South Asia from 1961 to 2018, however the productivity of ~3 t/ha is not sufficient to meet the future demands. Even though India had a record wheat production of 106 MMT in 2019-20, the Asian countries must cumulatively produce > 400 million tons of wheat by 2050. When compared to the highest productivity obtained in some of the Asian countries -Saudi Arabia (5.6 t/ha, all irrigated), China (4.7 t/ha, mostly irrigated) and Uzbekistan (4.5 t/ha, mostly irrigated)-, South Asian countries still need to increase the productivity of wheat through modern breeding approaches and agronomic practices [2].

In India, wheat is the second major crop and staple food after rice in terms of land allocation, production and daily dietary intake. In 1950-51, the total area under wheat was 9.75 million ha, with an average yield of 0.66 t/ha; total production was 6.46 million metric tons (MMT) and 34% of the area was irrigated [3]. In 1967-68, wheat yield in India for the first time exceeded a ton per ha (1.10 t/ha), with a total production of 16.5 MMT from nearly 15 million ha of land of which 43.4% was irrigated [3]. Despite the dramatic increase in yields, up to 1993 India was a net wheat importing country, with sporadic wheat exports [4]. By 2016-18 triennium average, wheat area of India reached 30.3 million ha of which more than 94% is irrigated, and with a yield of 3.2 t/ha, total wheat production was 96.8 MMT [3]. Currently, India is the second largest wheat-producing country in the world after China (131.4 million tons), and India’s production is nearly 14% of the total wheat in the world [5].

Interestingly, similar to Bangladesh, and other countries in Africa [6-8], the demand for wheat has been increasing in South Asia. Bangladesh is traditionally a rice growing country but became a wheat growing country through the wheat production program initiated in 1975, which is grown in rotation with rice. Currently, wheat is grown in ~2% of the total cultivated area and the demand for wheat is increasing [9]. However, Pakistan, where wheat is the largest crop and staple food, produces about 25 MMT of wheat. Improved semi-dwarf wheat cultivars available in Pakistan have genetic yield potential of 7-8 t/ha whereas national average yields are about 2.8 t/ha [10, 11]. Iran is generally considered a central Asian country, but the UN classification adds Iran also to South Asia. It is a semi-arid country where many crops originated and is close to the center of origin of wheat. Other South Asian countries that have wheat production are Afghanistan and Nepal.

The potential for agricultural extensification in the form of land expansion for wheat is very limited in S Asia, so cultivating climate resilient high yielding wheat can be the best way to enhance production to meet the growing demand. Climate resilient wheat is becoming more important for India due to global warming; the winter in India is becoming shorter and warmer, which has already generated threats for sustainable wheat production [12]. Therefore, the priority areas of research and development to increase wheat productivity are investments in hybrid wheat, exploitation of alien and related species, water use efficiency, high temperature tolerance and pyramiding of disease resistance [13].

## 2 EFFECTS OF CLIMATE CHANGE AND THE DEVELOPMENT OF CLIMATE RESILIENT WHEAT FOR SOUTH ASIA

Current trends in population growth suggest that global food production is unlikely to satisfy future demand under predicted climate change scenarios unless rates of crop improvement are accelerated. In order to maintain food security in the face of these challenges, a holistic approach that includes stress-tolerant germplasm, sustainable crop and natural resource management, and sound policy interventions will be needed [14]. In Europe, the current breeding programs and cultivar selection practices do not sufficiently prepare for climatic uncertainty and variability [15]. On the other hand, climate resilience research in Australia focuses on the likely impacts of increases in elevated carbon dioxide (CO_2_) and the variability of climate with the potential to increase the occurrence of abiotic stresses such as heat, drought, waterlogging, and salinity [16]. According to the Mega-Environment (ME) classification, most of South Asia (low rainfall irrigated ME1 (Indo Gangetic plans, Pakistan), residual moisture ME 4C (Indore, India), high rainfall humid hot 5A (Bangladesh) comes under the climate of high temperature, low rainfall areas [17] where the effects of climate change will be drastic.

Global wheat production is estimated to fall by on average 6% for each °C of further temperature increase and become more variable over space and time [18-20]. Simulations models predict that the future hot spots that will see a large reduction in grain yield are in India and Pakistan [21]. Cropping windows of the wheat-rice rotation will be affected by climate change, as predicted by simulation models, which in turn may reduce wheat yields [22]. In Pakistan, reduction in wheat phenological phases will be observed due to increased temperatures and thereby reduction in yield [23, 24]. In Nepal, the increased temperature also affects wheat production and net revenues negatively [25]. Raising temperatures and the resultant decreased cycle length will reduce grain yield in Bangladesh [26]. It was reported that a one percent increase in temperature decreases yield by over 2.5% in the northern region of Dinajpur in Bangladesh [27]. Similar negative trends will be observed in Afghanistan and Iran [28].

Delivering climate ready wheat will require the integration of different disciplines; pre-breeding and genomics assisted breeding can contribute to the more efficient development of climate resilient crops [29, 30]. High throughput phenotyping and genomic assisted breeding are key to developing tolerant wheat [31]. In addition, crop modelling can help in developing climate smart wheat [32]. Genetic variability is the very basis of any crop improvement program and wild relatives, landraces and genetic stocks are important sources for new genetic diversity. However, with the use of conventional breeding tools and approaches that focus largely on elite germplasm, genetic gains hover around 1% per year. Hence, there has not been any quantum yield gain in the new varieties, highlighting the need to complement conventional approaches with modern pre-breeding methods [1].

## 3 WHAT IS PRE BREEDING AND HOW PRE-BREEDING HELPS IN THE DEVELOPMENT OF CLIMATE RESILIENT WHEAT

**Fig. (1):**
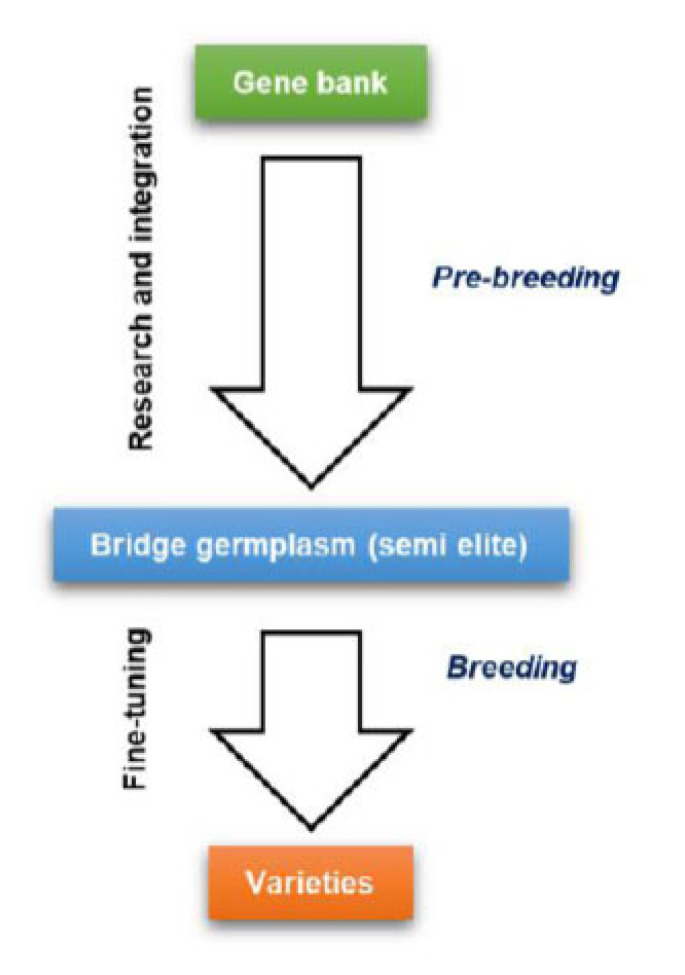
An illustration showing how pre-breeding and breeding differ in terms of efforts and length. (*A higher resolution / colour version of this figure is available in the electronic copy of the article*).

Pre-breeding is the introgression of desirable genes from exotic germplasm into genetic backgrounds readily usable by breeders with minimum linkage drag. Pre-breeding activities use promising landraces, wild relatives, and popular cultivars to transfer the desired alleles or traits into the elite background [33-36]. Whereas, breeding is more focused on crossing the best × best and selecting the best [37]. The widely practiced focus on ‘working collections’ with limited genetic diversity restricts the variability in cultivars. Hence, exploitation of the genetic diversity present in the gene banks will help to increase the diversity of the cultivars [38]. Generally, the process of pre-breeding is more time consuming when compared to breeding. Pre-breeding involves the research for traits, trait combinations, genetic basis of traits, marker development, predictive models, and germplasm exploration whereas breeding is more fine tuning of the best lines and their recombination for adaptation to specific environments (Fig. **1**).

### 3.1 Major Genetic Resources Available that Can Contribute to Pre-breeding Efforts in South Asia

#### 3.1.1 CIMMYT Gene Bank, Mexico

Wheat holdings at CIMMYT gene bank comprise of 150,000 seed samples from more than 100 countries; the largest unified collection in the world for a single crop. In 2012 and 2013, about 70,000 accessions of wheat were phenotyped for several agronomic traits in the hot desert of Sonora, Mexico to develop diversity and trait panels [39]. A recent genetic study also genetically characterized 80,000 accessions; 56,342 domesticated hexaploid, 18,946 domesticated tetraploid and 3,903 crop wild relatives from wheat gene bank using *DArTseq* markers [40]. CIMMYT gene bank has close to 2,000 wheat synthetic hexaploid lines. Among them, many were utilized for pre-breeding and breeding. A recent study revealed that in the bread wheat breeding program 20% of the lines were primary synthetic derived and well represented in international nurseries [41].

#### 3.1.2 National Bureau of Plant Genetic Resources (NBPGR), New Delhi

The NBPGR was established by the Indian Society of Genetics and Plant Breeding in a meeting in 1941 to conserve germplasm collection in India. Dr. B.P. Pal working at the Indian Agriculture Research Institute (IARI) approached the Imperial Council of Agricultural Research to set up a unit for assembly of global germplasm under phytosanitary conditions in India. The division of Botany at IARI, now known as Division of Genetics, started a separate scheme for ‘Plant Introduction’ under the leadership of Dr. Harbhajan Singh in 1970. Government of India upgraded it to an independent institute named as ‘National Bureau of Plant Introduction’ in August 1976 which was renamed as ‘National Bureau of Plant Genetic Resources’ (NBPGR) in January 1977. NBPGR is a nodal organization in India for the management of plant genetic resources. Here a total of 1762 crop species were conserved, it houses the second largest gene bank in the world with a total of 446,636 accessions collections which includes 5,034 Released Varieties and 4,316 Genetic Stocks apart from the exploration collections and introductions. *In Vitro* Gene bank holds 1902 collections and Cryogene bank with 11839 accessions.

NBPGR holds 34542 wheat accessions including 2304 collections of 55 wild species, 14702 exotic collections from different countries, 585 landraces, 338 genetic stocks and 428 released varieties from India. These had been assembled from different agro-ecological regions of India, and from 99 countries, mainly from South America and North America [42]. A rich diversity for wheat still found in India in the northern states of Himalayan region and also in hill regions. Semi-arid region of Rajasthan, Gujarat, Karnataka, which possesses drought, heat and salinity tolerant types wheat lines. It also holds the trial materials (10,771) received from international organizations and other collaborative research works. At National Active Germplasm Sites for active collections at Indian Institute of Wheat and Barley Research Institute Karnal holds 7,000 active germplasm collections which available for all the breeders across the country.

In India, a comprehensive germplasm evaluation and characterization study was conducted for wheat accessions conserved in the Indian National Gene bank to identify sources of resistance/tolerance to different biotic and abiotic stresses. Gene bank accessions comprising three species of wheat *Triticum aestivum*, *T*. *durum* and *T*. *dicoccum* were screened sequentially at multiple disease hotspots, during the 2011–14 crop seasons, carrying only resistant accessions to the next step of evaluation a core set and to develop a trait-specific reference sets were assembled and detailed characterization reports were available for these sets [43]. These accessions also need to be screened for abiotic stress tolerance to develop germplasm for climate resilience.

#### 3.1.3 National Plant Gene Bank-Iran

This was founded in 1983 and holds 155 species of crop plants. Several wheat accessions from this gene bank are also available at CIMMYT gene bank. Recent studies used the availability of Iranian wheat landraces at CIMMYT for genomic prediction and deployment of them into breeding [44, 45].

#### 3.1.4 Gene Bank in Bangladesh, Pakistan, Nepal, Afghanistan

Bangladesh wanted to establish gene bank for several species and efforts started as early as 2012 (http://www.isaaa.org/kc/cropbiotechupdate/article/default.asp?ID=9982). As far as we know, other south Asian countries do not have a gene bank that holds several thousand wheat accessions.

### 3.2 Modern Pre-breeding Methods in Practice

#### 3.2.1 Trait Based Crossing and Generation Advancement

**Fig. (2):**
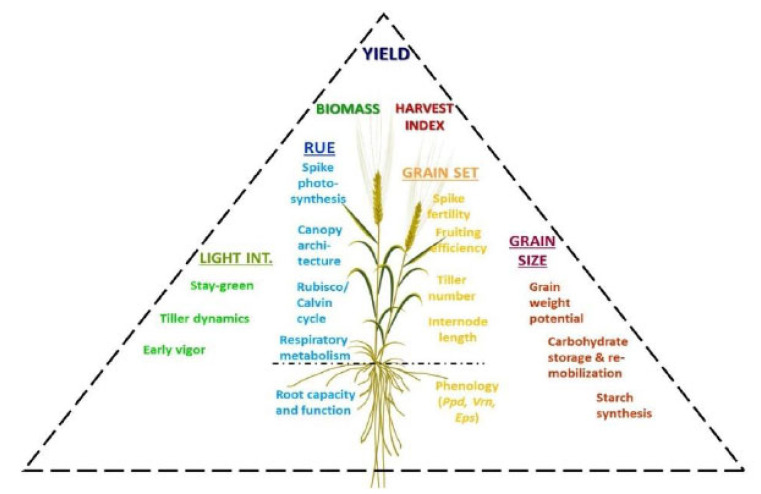
Trait hierarchy based on dissecting grain yield into biomass and harvest index components and sub-traits adapted from Breeder Friendly Phenotyping [50]. (*A higher resolution / colour version of this figure is available in the electronic copy of the article*).

The physiological pre-breeding approach focuses on dissecting complex traits -like heat or drought adaptation- into component traits -like deep rooting and storage of reserve carbohydrates- and making complementary crosses to increase the grain yield under abiotic stress conditions [46, 47]. This involves dissecting yield into its two main drivers, biomass and harvest index [48, 49] and identifying genetic markers and proxy traits for them (Fig. **2**). Most of the complex traits cannot be dissected into simple molecular markers since the percentage of variation explained by any of the many molecular markers identified is not close to the heritability estimates. However, screening advanced generations using high throughput methods and field based phenotyping have proven to improve the selection for high yielding lines [50].

Trait based pre-breeding for heat stress tolerance is based on the conceptual model for heat stress tolerance; where,

Grain yield = LI × RUE × HI (1) 

LI is light interception, RUE is radiation use efficiency, and HI is harvest index [51]. In short, the traits that are of interest to increase heat stress tolerance can be divided into partitioning (HI), Light Interception (LI), Water Use (WU), and photo protection. The RUE traits are leaf morphology, wax, pigments such as chlorophyll *a:b*, carotenoids, antioxidants, CO_2_ fixation traits (Rubisco specificity and rubisco activase), spike photosynthesis, membrane thermostability, and respiration. Traits related to HI include pollen sterility, floret survival, grain filling, and stem carbohydrate storage and remobilization. LI is related to rapid growth cover and stay green. WUE is related to root traits and transpiration regulated by plant signaling [48].

Yield under drought can be also be shown using conceptual models,

Grain yield = WU × WUE × HI (2)

Where WU is water use, WUE is water use efficiency, and HI is harvest index. Here, water use is dependent on water uptake by roots. Main traits of importance to improve drought stress tolerance includes leaf morphology, leaf rolling, wax, pubescence, spike/awn photosynthesis, harvest index, biomass, stem carbohydrates, early vigor and ground cover, length of coleoptile, seed size, leaf water content, canopy temperature, and root system [39, 51, 52]. These traits are screened in different diversity and trait panels to initiate complementary crosses and selection for improved yield under drought and heat stress conditions [46].

#### 3.2.2 Marker Assisted Crossing and Generation Advancement

Use of markers for breeding can be classified into two groups based on the genetic structure of traits (1) simple traits and (2) complex traits [53]. Simple traits are those governed by few genes (*e.g.* Flowering time, plant height), whereas complex traits are governed by many QTL and the G × E are high for them compared to simple traits. Use of markers in pre-breeding involves understanding the genetic basis of traits, marker discovery and development of usable markers, integration of the trait into elite lines through marker assisted selection or back crossing, pyramiding traits through genomic prediction or trait-based stacking. Among above mentioned traits used in pre-breeding canopy temperature and stay green has been widely studied and genetic basis was explored [54, 55]. Recent studies have also focused on understanding the genetic basis of biomass and harvest index, two-component traits of grain yield. Many studies have identified QTL for several physiological and agronomic traits, but few have resulted in their use in pre-breeding. Major genes of phenology traits are not routinely used in pre-breeding since they are easy to measure in many lines. However, complex traits which are difficult to measure are high priorities for the development of markers are use in selection of segregating generations. The most common marker that is used in selection is for thousand grain weight in CIMMYT, since it is a high heritability trait and KASP markers are available [56-58]. For complex traits where marker traits associations (MTAs) or QTLs that explain higher percentage of variation of the traits is not available, phenomics based selection or the application of genomic prediction might help in pre-breeding [59].

#### 3.2.3 Genomic Tools for Pre-Breeding

Wheat has a huge genome (16Gb) among crop plants with similarities between sub-genomes and abundant repetitive elements (85% of the genome). Recent advances in wheat genome sequencing may influence the development of high yielding lines under abiotic stresses. It may help to identify novel alleles and gene cloning in wheat for important traits. A pan-genome analysis has shown introgressions from wild wheat for adapting to diverse environments [60]. In addition, it may help to identify haplotypes and haplotype blocks for breeding. Another approach is based on gene editing which can be used to create a new genetic variation to study as well as to edit genes to fit future climate. A study on grain weight has shown the potential of gene editing in wheat [61].

#### 3.2.4 Physiological Pre-breeding and its Outputs

**Fig. (3):**
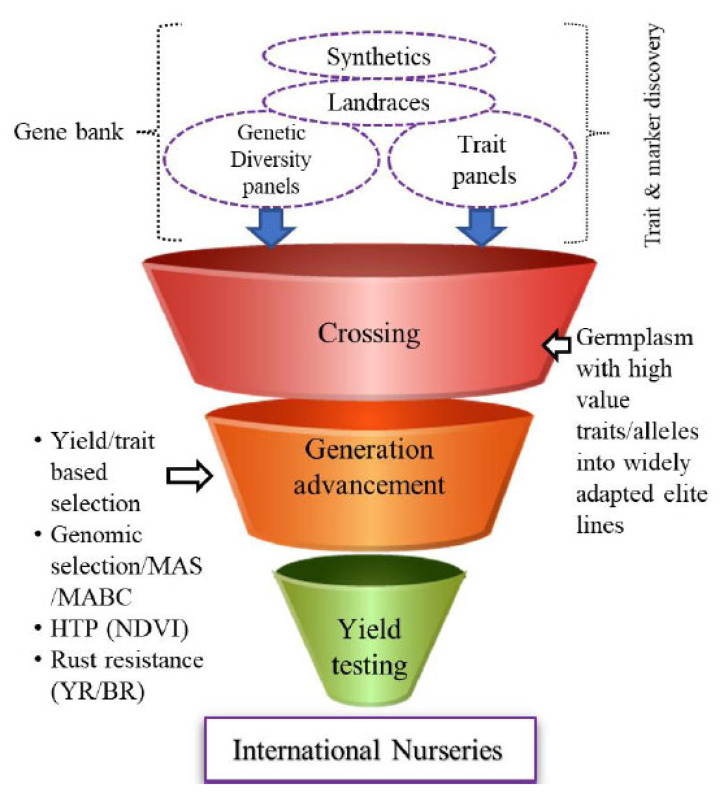
An illustration of CIMMYT’s successful pre-breeding pipeline for abiotic stress tolerance adapted from [69]. (*A higher resolution / colour version of this figure is available in the electronic copy of the article*).

Pre-breeding involves stacking different traits and alleles that are complementary to increase the desired traits in elite germplasm, and thereby grain yield (Liu *et al.*, 2020). Several genetic and physiological approaches are followed in pre-breeding. The germplasm mainly used are synthetics and landraces, which are crossed and backcrossesd to widely adapted elite lines. The resulting generations are screened based on different traits and markers to select the best fixed lines for specific traits that are associated with high yield under abiotic stress (Fig. **3**). High throughput phenotyping for canopy temperature and Normalized Difference Vegetation Index (NDVI), genomic prediction and selection for grain yield, screening for rust resistance; yellow and brown rust resistance are routinely conducted in pre-breeding. Pre-breeding also involves several of the initial steps of a breeding program.

CIMMYT’s physiology pre-breeding populates special nurseries of spring wheat that are tolerant to heat, and drought stress called *Stress Adapted Trait Yield Nurseries* (SATYNs) (Table **1**). Each alternate SATYNs are for drought or heat stress *e.g*. 1, 3, 5, and 7 SATYNs have germplasm that has drought stress tolerance when compared to checks, whereas lines in 2, 4, 6, and 8^th^ SATYNs have heat stress tolerance. Up to now, 272 lines were distributed to 5 countries in south Asia and 29 locations through SATYNs. However, if we consider the locations around the world 499 collaborators received the SATYNs (Table **2**). These special nurseries have lines developed by the crossing of synthetics or a landrace with high value allele/traits into an elite background. They are grown by the national and private breeders in several countries in South Asia; India, Pakistan, Nepal, Bangladesh, Iran, and Afghanistan. Even though the main purpose of these SATYN are to provide semi-elite germplasm for use as parents to develop heat /drought tolerance varieties, some of the lines from physiological pre-breeding have resulted in direct release as varieties in west of India (Table **3**). These releases have a Mexican landrace or a synthetic wheat in their pedigree.

## 4 PROSPECTS OF DEVELOPING CLIMATE RESILIENT WHEAT THROUGH PRE-BREEDING

**Fig. (4):**
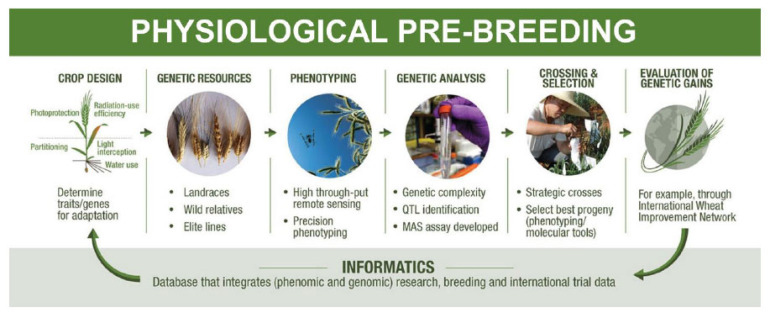
Physiological pre-breeding pipeline in CIMMYT adapted from [46]. (*A higher resolution / colour version of this figure is available in the electronic copy of the article*).

Pre-breeding is important in a crop like wheat which provides 20% of calories and proteins to the world population. Any major catastrophe will jeopardize the food supply of the >7 billion population. The genetic bottle neck created by the continuous use of working collections of advanced lines needs to be complemented through pre-breeding efforts. However, the huge number of germplasms present in the gene bank and linkage drag currently limit the utilization of these resources. One approach to overcome that bottleneck is through physiological pre-breeding (Fig. **4**), where complementary crosses are made to improve the grain yield under drought and heat stress conditions using carefully selected genetic resources to reduce linkage drag [46, 47]. The second approach to utilize gene bank accessions is through predictive approaches using genomic prediction, where instead of growing the complete collection, a small subset can be genotyped and phenotyped for several traits and that can be used as training set to predict and test the gene bank accessions iteratively [62]. Both approaches need high quality phenotyping, including remote sensing and precision phenotyping [50].

A recent study has shown the environment in Cd. Obregon where CIMMYT has it major research station is very important to develop lines for South Asia [63]. The success of pre-breeding done at the dessert in Cd. Obregon, Mexico for South Asia is due to the high correlation of grain yield in several traits conducted in Mexico and India [64]. Therefore, continued supply for improved germplasm from CIMMYT gene bank through physiological pre-breeding approach is one of the most important new approaches. An example of the germplasm provided with associated information -phenotypic data under irrigated, drought, and heat stress conditions and molecular marker profile of each line for major genes related to flowering time, plant height, vernalization, and rust resistance genes are shown in Supplementary Table **S1**.

In India, the main challenges of wheat productivity enhancement are climate change, sinking land and water resources, lack of pre-breeding efforts, in addition to the major breeding constraints of narrow genetic base, lack of phenotyping methods [65]. However, India has released many varieties for abiotic stress conditions compared to other countries that benefit farmers (Table **4**). They are specifically suited to Northwestern plain zone (NWPZ), central zone (CZ), peninsular zone (PZ), and northwest zone (NW) [66]. In addition, there is a lot of scope for a more internationally coordinated approach to crop phenotyping and modeling, combined with effective sharing of knowledge, facilities, and data, will boost the cost effectiveness and facilitate genetic gains of all staple crops, with likely spill over to more neglected crops [67]. Therefore, recent consortiums such as HeDWIC (http://www.hedwic.org/) that facilitate global coordination of wheat research to adapt to a future with more severe weather extremes, specifically heat and drought, are timely.

## CONCLUSION

HeDWIC delivers new technologies to wheat breeders worldwide *via* the International Wheat Improvement Network (IWIN), coordinated for more than half a century by the International Maize and Wheat Improvement Center.

## Figures and Tables

**Table 1:** Number of site and locations where SATYN nurseries were grown in South Asia from 2013 to 2019.

**SATYN** **Nursery**	**Traits and Alleles for Tolerance**	**No.** **of Lines**	**No. of Partners to which SATYN was Distributed**	**Countries and No. of Locations that Returned Data from South Asia**
1^st^ SATYN	Drought stress	68	9	India (4), Pakistan (2), Bangladesh (1), Nepal (1)
2^nd^ SATYN	Heat stress	37	23	India (6), Pakistan (3), Bangladesh (2), Nepal (1), Iran (2)
3^rd^ SATYN	Drought stress	24	55	India (8), Pakistan (4), Bangladesh (3), Nepal (1), Iran (4)
4^th^ SATYN	Heat stress	28	55	India (8), Pakistan (5), Nepal (1), Iran (4)
5^th^ SATYN	Drought stress	35	47	India (4), Pakistan (2), Nepal (1), Bangladesh (2), Iran (4)
6^th^ SATYN	Heat stress	26	105	India (4)
7^th^ SATYN	Drought stress	27	102	India (7), Pakistan (3), Nepal (1), Bangladesh (1), Iran (3)
8^th^ SATYN	Heat stress	27	103	-

**Table 2:** Some of the best lines from SATYNs based on statistical analysis of the global data that were distributed to South Asia.

**GID**	**Cross Name**	**SATYN**	**Trait**
6056139	*SOKOLL/WBLL1	1^st^ SATYN	Drought stress tolerance
6056165	WBLL4//***OAX93.24.35***/WBLL1	1^st^ SATYN	Drought stress tolerance
7142186	KS940935.7.1.2/2*PASTOR/4/FRAME//MILAN/KAUZ/3/PASTOR	4^th^ SATYN	Heat stress tolerance
6676763	**SOKOLL**/WBLL1	4^th^ SATYN	Heat stress tolerance
7129693	SERI/BAV92//***PUB94.15.1.12***/WBLL1	6^th^ SATYN	Heat stress tolerance
8188467	***IRAN-880***/3/2*ATTILA*2/PBW65//MURGA	7^th^ SATYN	Drought stress tolerance
6676793	**SOKOLL**/WBLL1	7^th^ SATYN	Drought stress tolerance
5865670	BAV92/SERI	8^th^ SATYN	Heat stress tolerance
7032370	JNRB.5/PIFED/5/BJY/COC//PRL/BOW/3/SARA/THB//VEE/4/PIFED	8^th^ SATYN	Heat stress tolerance
7705584	***MEX94.27.1.20***/3/SOKOLL//ATTILA/3*BCN/4/PUB94.15.1.12/WBLL1	8^th^ SATYN	Heat stress tolerance
6056064	***PUB94.15.1.12***/WBLL1	8^th^ SATYN	Heat stress tolerance
7705129	**SOKOLL**/3/PASTOR//HXL7573/2*BAU/4/PARUS/PASTOR	8^th^ SATYN	Heat stress tolerance
8329091	**SOKOLL**/5/W15.92/4/PASTOR//HXL7573/2*BAU/3/WBLL1/6/SOKOLL/3/PASTOR//HXL7573/2*BAU	8^th^ SATYN	Heat stress tolerance
6056140	**SOKOLL**/WBLL1	8^th^ SATYN	Heat stress tolerance
5865676	W15.92/4/PASTOR//HXL7573/2*BAU/3/WBLL1	8^th^ SATYN	Heat stress tolerance
8101631	68.111/RGB-U//WARD/3/FGO/4/RABI/5/AE.SQUARROSA (778)/7/2*CHWL86/6/FILIN/IRENA/5/CNDO/R143//ENTE/MEXI_2/3/AEGILOPS SQUARROSA (TAUS)/4/WEAVER	9^th^ SATYN	Drought stress tolerance
8101711	CETA/AE.SQUARROSA (435)/5/2*UP2338*2/SHAMA/3/MILAN/KAUZ//CHIL/CHUM18/4/UP2338*2/SHAMA	9^th^ SATYN	Drought stress tolerance
8198445	CHEN/AE.SQ//2*OPATA/3/FINSI/5/W15.92/4/PASTOR//HXL7573/2*BAU/3/WBLL1	9^th^ SATYN	Drought stress tolerance
8198432	**SOKOLL**/WBLL1/4/PASTOR//HXL7573/2*BAU/3/WBLL1	9^th^ SATYN	Drought stress tolerance

*The accessions in bold are synthetic derivatives, the accession in bold italics are landraces, and the accessions shaded are synthetics.

**Table 3:** Germplasm released as a variety in South Asia from physiological pre-breeding by the national agriculture research system (NARS) partners.

**Year**	**Name**	**Cross / Pedigree**	**Country**
2013	Pakistan-13	MEX94.27.1.20/3/SOKOLL//ATTILA/3*BCN	*Pakistan*
2016	Borlaug-16	SOKOLL/3/PASTOR//HXL7573/2*BAU	*Pakistan*
2017	Kohat 17	SOKOLL/WEEBIL	*Pakistan*
2020	Kunar 20	MEX94.27.1.20/3/SOKOLL//ATTILA/3*BCN/4/PUB94.15.1.12/WBLL1	*Afghanistan*

MEX94.27.1.20 is a landrace, Sokoll is a synthetic derivative line.

**Table 4:** Some of the varieties released in India from the national program suitable for abiotic stress conditions for the central zone (CZ), north west zone (NW), north eastern plain zone (NEPZ), and peninsular zone (PZ).

**Variety**	**Year**	**Production** **Condition**	**Parentage**	**Average Yield**	**Potential Yield**	**Notification No.**
HI 1634	2021	Irrigated, late sown conditions of CZ	GW 322/PBW 498	5.16	7.06	500(E)2/2/2021
HD3271	2020	Irrigated, very late Sown conditions of NW and NEPZ	Chiriya7 / HD2824	3.25	5.9	9(E) 06.01.2020
DBW187	2019	Irrigated, timely sown conditions of NEPZ	NAC/TH.AC//3*PVN/3/MIRLO/BUC/4/2*PASTOR/5/KACHU/6/KACHU	4.88	6.47	1498(E)01/04/2019
HD 3237	2019	Restricted irrigation, timely sown conditions of NEPZ	HD3016/HD2967	4.84	6.31	1498(E)01.04.2019
HI 8759	2016	Irrigated, timely sown conditions of CZ	HI 8663/HI 8498	5.69	6.43	100(E)30/3/2017
HS 562	2016	Rainfed & irrigated Timely sown conditions of NHZ	OASIS/SKAUZ//4*BCN/3/2*PASTOR	3.68/5.27	5.886.22	2238(E)29/06/2016
HD3086	2014	Irrigated, timely sown conditions of NW and NEPZ	DBW14/HD2733//HUW468	5.46	7.10	244(E)24/1/2014
KRL 210	2012	Saline and alkaline soil conditions of all the zones	PBW65/2*PASTOR	3.37	4.93	456(E)16.03.2012
HD 2967	2011	Irrigated, timely sown conditions of NW and NEPZ	ALD/COC//URES/HD2160M/HD3328	5.11	6.2	2326(E)1/10/2011
HD2987	2011	Rainfed & restricted irrigation, timely sown conditions of CZ	HI1011/HD2348//MENDOS//IWP72/DL153-2	1.75/3.15	3.22/3.86	632(E)25/3//2011
MACS 6222	2010	Irrigated, timely Sown of PZ	HD 2189*2//MACS2496	4.77	6.09	733(E)01.04.2010
HD2932	2008	Irrigated, late sown conditions of CZ and PZ	KAUZ/STAR//HD2643	4.20/4.33	5.78/5.36	1108(E)08.05.2008
HI1544	2008	Irrigated, timely sown conditions of CZ	HINDI62/BOBWHITE/CPAN 2099	5.14	6.82	1108(E)08.05.2008
HI1531	2006	Rainfed/restricted irrigation, timely sown conditions of CZ	HI 1182/CPAN 1990	2.4/2.7	3.98/4.00	599(E)25.04.2006
HI1500	2003	Rainfed, timely sown conditions of CZ	HW 2002*2//STREMPALLI/PNC5	1.6	3.00	283(E)12.03.2003
RAJ3765	1996	Irrigated, late Sown conditions of NWPZ	HD 2402/VL639	3.65	4.38	1(E)01.01.1996
Lok1	1982	Irrigated, timely sown and late Sown of CZ and PZ	S308/S331	3.8	4.54	19(E)14.01.1982
C306	1969	Rainfed timely sown conditions of NW and NEPZ	RGN/CSK3//2*C591/3/C217/N14//C281	2.6	3.6	4045(E)24.09.1969

## LIST OF ABBREVIATIONS

SATYN: Atress Adapted Trait Yield Nurseries
NARS: National Agricultural Research System
MMT: Million Metric Tons
QTL: Quantitative Trait Loci
NDVI: Normalized Difference Vegetative Index
CT: Canopy Temperature

## SUPPLEMENTARY MATERIAL

Supplementary material is available on the publisher’s website along with the published article. Supplementary Table **S1**. An example of associated data with the most recent SATYN distributed to many developing countries.

## References

[1] Paroda R., Dasgupta S., Mal B., Singh S.S., Jat M.L., Singh G. (2013). Proceedings of the regional consultation on improving wheat productivity in Asia.

[2] Braun H. (2012). Regional scenario of wheat in asia..

[3] Government of India (2019). Agricultural Statistics as a Glance 2018.

[4] FAOSTAT (2020). Crops and livestock products-trade.. Crops and livestock products.

[5] FAO (2020). Data: Production.. Online database on crop production, yield and harvested area.

[6] Mottaleb K.A., Rahut D.B., Kruseman G., Erenstein O. (2018). Evolving food consumption patterns of rural and urban households in developing countries.. Br. Food J..

[7] Mottaleb K.A., Rahut D.B., Kruseman G., Erenstein O. (2018). Changing food consumption of households in developing countries: A bangladesh case.. J. Int. Food Agribus. Mark..

[8] Mason N.M., Jayne T.S., Shiferaw B. (2015). Africa’s rising demand for wheat : Trends, drivers, and policy implications.. Dev. Policy Rev..

[9] Uddin M.J., Barma N.C.D., Sarker Z.I., Bodruzzama; Hakim P.K.M.M.A.A., Hossain M. I. (2012). Wheat production for food security in bangladesh..

[10] Abid S. (2019). Trends and variability of wheat crop in Pakistan.. Asian J. Agric. Rural Dev..

[11] Ali Chandio A., Jiang Y., Ali Joyo M., Rehman A. (2016). Impact of area under cultivation, water availability, credit disbursement, and fertilizer off-take on wheat production in pakistan.. J. Appl. Environ. Biol. Sci.

[12] Joshi A.K., Mishra B., Chatrath R., Ortiz Ferrara G., Singh R.P. (2007). Wheat improvement in India: Present status, emerging challenges and future prospects.. Euphytica.

[13] Rajaram S. (2013). Strategy for increasing wheat productivity..

[14] Braun H-J., Atlin G., Payne T., Reynolds M.P. (2010). Climate Change and Crop Production.

[15] Kahiluoto H., Kaseva J., Balek J., Olesen J.E., Ruiz-Ramos M., Gobin A., Kersebaum K.C., Takáč J., Ruget F., Ferrise R., Bezak P., Capellades G., Dibari C., Mäkinen H., Nendel C., Ventrella D., Rodríguez A., Bindi M., Trnka M. (2019). Decline in climate resilience of European wheat.. Proc. Natl. Acad. Sci. USA.

[16] Chapman S.C., Chakraborty S., Dreccer M.F., Howden S.M. (2012). Plant adaptation to climate changeopportunities and priorities in breeding.. Crop Pasture Sci..

[17] Braun H-J., Rajaram S., van Ginkel M. (1996). CIMMYT’s approach to breeding for wide adaptation.. Euphytica.

[18] Asseng S. (2014). Rising temperatures reduce global wheat production.. Nat. Clim. Chang..

[19] Liu B. (2016). Similar estimates of temperature impacts on global wheat yield by three independent methods.. Nat. Clim. Chang..

[20] Zhao C., Liu B., Piao S., Wang X., Lobell D.B., Huang Y., Huang M., Yao Y., Bassu S., Ciais P., Durand J.L., Elliott J., Ewert F., Janssens I.A., Li T., Lin E., Liu Q., Martre P., Müller C., Peng S., Peñuelas J., Ruane A.C., Wallach D., Wang T., Wu D., Liu Z., Zhu Y., Zhu Z., Asseng S. (2017). Temperature increase reduces global yields of major crops in four independent estimates.. Proc. Natl. Acad. Sci. USA.

[21] Asseng S., Cammarano D., Basso B., Chung U., Alderman P.D., Sonder K., Reynolds M., Lobell D.B. (2017). Hot spots of wheat yield decline with rising temperatures.. Glob. Change Biol..

[22] Sandhu S.S., Kaur P., Gill K.K., Vashisth B.B. (2019). The effect of recent climate shifts on optimal sowing windows for wheat in Punjab, India.. J. Water Clim. Chang..

[23] Ali S., Liu Y., Ishaq M., Shah T., Abdullah,; Ilyas A., Din I.U. (2017). Climate change and its impact on the yield of major food crops: Evidence from pakistan.. Foods.

[24] Ahmad S. (2019). Climate warming and management impact on the change of phenology of the rice-wheat cropping system in Punjab, Pakistan.. F. Crop. Res..

[25] Thapa-Parajuli R., Devkota N. (2016). Impact of climate change on wheat production in Nepal.. Asian J. Agric. Extension Econ. Sociol..

[26] Hasan M.M., Alauddin M., Rashid Sarker M.A., Jakaria M., Alamgir M. (2019). Climate sensitivity of wheat yield in Bangladesh: Implications for the United Nations sustainable development goals 2 and 6.. Land Use Policy.

[27] Syeda J. (2018). Impact of climate change on wheat production in dinajpur region of bangladesh: An econometric analysis.. J. Environ. Sci. Nat. Resour..

[28] Karimi V., Karami E., Keshavarz M. (2018). Climate change and agriculture: Impacts and adaptive responses in Iran.. J. Integr. Agric..

[29] Varshney R.K., Singh V.K., Kumar A., Powell W., Sorrells M.E. (2018). Can genomics deliver climate-change ready crops?. Curr. Opin. Plant Biol..

[30] Ortiz R., Reynolds M. P. (2010). Adapting crops to climate change: A summary.

[31] Juliana P. (2018). Integrating genomic - enabled prediction and high - throughput phenotyping in breeding for climate - resilient bread wheat.. Theor. Appl. Genet..

[32] Ramirez-Villegas J. (2020). CGIAR modeling approaches for resource- constrained scenarios: I. Accelerating crop breeding for a changing climate.. Crop Sci..

[33] Sharma S. (2017). Prebreeding using wild species for genetic enhancement of grain legumes at ICRISAT.. Crop Sci..

[34] Nass L.L., Paterniani E. (2000). Pre-breeding : A link between genetic resources and maize breeding.. Sci. Agric..

[35] Stander J.R. (1993). Pre-breeding from the perspective of the private plant breeder.. J. Sugar Beet Res..

[36] Singh K., Kumar S., Kumar S.R., Singh M., Gupta K. (2019). Plant genetic resources management and pre-breeding in genomics era.. Indian J. Genet. Plant Breed..

[37] Van Ginkel M., Ortiz R., Van Ginkel M., Ortiz R. (2018). Cross the best with the best, and select the best: HELP in breeding selfing crops.. Crop Sci..

[38] Moore G. (2015). Strategic pre-breeding for wheat improvement.. Nat. Plants.

[39] Reynolds M., Tattaris M., Cossani C.M., Ellis M., Yamaguchi-Shinozaki K., Saint Pierre C. (2015). Adv. Wheat Genet. From Genome to F.

[40] Sansaloni C., Franco J., Santos B., Percival-Alwyn L., Singh S., Petroli C., Campos J., Dreher K., Payne T., Marshall D., Kilian B., Milne I., Raubach S., Shaw P., Stephen G., Carling J., Pierre C.S., Burgueño J., Crosa J., Li H., Guzman C., Kehel Z., Amri A., Kilian A., Wenzl P., Uauy C., Banziger M., Caccamo M., Pixley K. (2020). Diversity analysis of 80,000 wheat accessions reveals consequences and opportunities of selection footprints.. Nat. Commun..

[41] Rosyara U., Kishii M., Payne T., Sansaloni C.P., Singh R.P., Braun H.J., Dreisigacker S. (2019). Genetic contribution of synthetic hexaploid wheat to cimmyt’s spring bread wheat breeding germplasm.. Sci. Rep..

[42] Phogat B.S. (2020). Characterization of wheat germplasm conserved in the Indian National Genebank and establishment of a composite core collection.. Crop Sci..

[43] Kumar S., Archak S., Tyagi R.K., Kumar J., Vk V., Jacob S.R., Srinivasan K., Radhamani J., Parimalan R., Sivaswamy M., Tyagi S., Yadav M., Kumari J., Deepali, Sharma S., Bhagat I., Meeta M., Bains N.S., Chowdhury A.K., Saha B.C., Bhattacharya P.M., Kumari J., Singh M.C., Gangwar O.P., Prasad P., Bharadwaj S.C., Gogoi R., Sharma J.B., Gm S.K., Saharan M.S., Bag M., Roy A., Prasad T.V., Sharma R.K., Dutta M., Sharma I., Bansal K.C. (2016). Evaluation of 19,460 wheat accessions conserved in the indian national genebank to identify new sources of resistance to rust and spot blotch diseases.. PLoS One.

[44] Sehgal D., Vikram P., Sansaloni C.P., Ortiz C., Pierre C.S., Payne T., Ellis M., Amri A., Petroli C.D., Wenzl P., Singh S. (2015). Exploring and mobilizing the gene bank biodiversity for wheat improvement.. PLoS One.

[45] Crossa J. (2016). Genomic prediction of gene bank wheat landraces.. G3 Genes|Genomes|Genetics.

[46] Reynolds M., Langridge P. (2016). Physiological breeding.. Curr. Opin. Plant Biol..

[47] Reynolds M.P. (2017). Strategic crossing of biomass and harvest index—source and sink—achieves genetic gains in wheat.. Euphytica.

[48] Cossani C.M., Reynolds M.P. (2012). Physiological traits for improving heat tolerance in wheat.. Plant Physiol..

[49] Molero G., Joynson R., Pinera-Chavez F.J., Gardiner L.J., Rivera-Amado C., Hall A., Reynolds M.P. (2019). Elucidating the genetic basis of biomass accumulation and radiation use efficiency in spring wheat and its role in yield potential.. Plant Biotechnol. J..

[50] Reynolds M., Chapman S., Crespo-Herrera L., Molero G., Mondal S., Pequeno D.N.L., Pinto F., Pinera-Chavez F.J., Poland J., Rivera-Amado C., Saint Pierre C., Sukumaran S. (2020). Breeder friendly phenotyping.. Plant Sci..

[51] Reynolds M. P., Saint Pierre C., Saad A. S. I., Vargas M., Condon A. G. (2007). Evaluating potential genetic gains in wheat associated with stress-adaptive trait expression in elite genetic resources under drought and heat stress.. Crop Sci..

[52] Reynolds M.P., Mujeeb-Kazi A., Sawkins M. (2005). Prospects for utilising plant-adaptive mechanisms to improve wheat and other crops in drought- and salinity-prone environments.. Ann. Appl. Biol..

[53] Falconer D. S., Mackay T. F. C. (1961). Introduction to quantitative genetics.

[54] Pinto R.S., Reynolds M.P., Mathews K.L., McIntyre C.L., Olivares-Villegas J.J., Chapman S.C. (2010). Heat and drought adaptive QTL in a wheat population designed to minimize confounding agronomic effects.. Theor. Appl. Genet..

[55] Pinto R.S., Lopes M.S., Collins N.C., Reynolds M.P. (2016). Modelling and genetic dissection of staygreen under heat stress.. Theor. Appl. Genet..

[56] Simmonds J., Scott P., Leverington-Waite M., Turner A.S., Brinton J., Korzun V., Snape J., Uauy C. (2014). Identification and independent validation of a stable yield and thousand grain weight QTL on chromosome 6A of hexaploid wheat (Triticum aestivum L.).. BMC Plant Biol..

[57] Brinton J., Simmonds J., Minter F., Leverington-Waite M., Snape J., Uauy C. (2017). Increased pericarp cell length underlies a major quantitative trait locus for grain weight in hexaploid wheat.. New Phytol..

[58] Simmonds J., Scott P., Brinton J., Mestre T.C., Bush M., Del Blanco A., Dubcovsky J., Uauy C. (2016). A splice acceptor site mutation in TaGW2-A1 increases thousand grain weight in tetraploid and hexaploid wheat through wider and longer grains.. Theor. Appl. Genet..

[59] Sukumaran S., Crossa J., Jarquin D., Lopes M., Reynolds M. P. (2017). Genomic prediction with pedigree and genotype × environment interaction in spring wheat grown in south and west asia, north africa, and mexico.. G3 Genes|Genomes|Genetics.

[60] Walkowiak S., Gao L., Monat C., Haberer G., Kassa M.T., Brinton J., Ramirez-Gonzalez R.H., Kolodziej M.C., Delorean E., Thambugala D., Klymiuk V., Byrns B., Gundlach H., Bandi V., Siri J.N., Nilsen K., Aquino C., Himmelbach A., Copetti D., Ban T., Venturini L., Bevan M., Clavijo B., Koo D.H., Ens J., Wiebe K., N’Diaye A., Fritz A.K., Gutwin C., Fiebig A., Fosker C., Fu B.X., Accinelli G.G., Gardner K.A., Fradgley N., Gutierrez-Gonzalez J., Halstead-Nussloch G., Hatakeyama M., Koh C.S., Deek J., Costamagna A.C., Fobert P., Heavens D., Kanamori H., Kawaura K., Kobayashi F., Krasileva K., Kuo T., McKenzie N., Murata K., Nabeka Y., Paape T., Padmarasu S., Percival-Alwyn L., Kagale S., Scholz U., Sese J., Juliana P., Singh R., Shimizu-Inatsugi R., Swarbreck D., Cockram J., Budak H., Tameshige T., Tanaka T., Tsuji H., Wright J., Wu J., Steuernagel B., Small I., Cloutier S., Keeble-Gagnère G., Muehlbauer G., Tibbets J., Nasuda S., Melonek J., Hucl P.J., Sharpe A.G., Clark M., Legg E., Bharti A., Langridge P., Hall A., Uauy C., Mascher M., Krattinger S.G., Handa H., Shimizu K.K., Distelfeld A., Chalmers K., Keller B., Mayer K.F.X., Poland J., Stein N., McCartney C.A., Spannagl M., Wicker T., Pozniak C.J. (2020). Multiple wheat genomes reveal global variation in modern breeding.. Nature.

[61] wang e. wei (2018). Gene editing and mutagenesis reveal inter-cultivar differences and additivity in the contribution of TaGW2 homoeologues to grain size and weight in wheat.. bioRxiv.

[62] Yu X., Li X., Guo T., Zhu C., Wu Y., Mitchell S.E., Roozeboom K.L., Wang D., Wang M.L., Pederson G.A., Tesso T.T., Schnable P.S., Bernardo R., Yu J. (2016). Genomic prediction contributing to a promising global strategy to turbocharge gene banks.. Nat. Plants.

[63] Juliana P., Singh R.P., Braun H.J., Huerta-Espino J., Crespo-Herrera L., Payne T., Poland J., Shrestha S., Kumar U., Joshi A.K., Imtiaz M., Rahman M.M., Toledo F.H. (2020). Retrospective quantitative genetic analysis and genomic prediction of global wheat yields.. Front. Plant Sci..

[64] Sukumaran S., Lopes M., Dreisigacker S., Reynolds M. (2018). Correction to: Genetic analysis of multi-environmental spring wheat trials identifies genomic regions for locus-specific trade-offs for grain weight and grain number.. Theor. Appl. Genet..

[65] Bansal K. C., Tyagi R. K., Dutta M., Phogat B. S., Archak S. (2013). Strategies for enhanced utilization of *ex situ* wheat genetic resources to boost wheat productivity: Indian initiatives..

[66] Gupta A. (2018). Wheat varieties notified in India since 1965.

[67] Reynolds M.P. (2016). An integrated approach to maintaining cereal productivity under climate change.. Glob. Food Secur..

[68] Reynolds M., Sukumaran S., Pinto F., Molero G. (2020). Population, agriculture, and biodiversity: Problems and prospects.

